# EARLY BUD BREAK 1 triggers bud break in peach trees by regulating hormone metabolism, the cell cycle, and cell wall modifications

**DOI:** 10.1093/jxb/eraa119

**Published:** 2020-03-09

**Authors:** Xuehui Zhao, Xiaolun Han, Qingjie Wang, Xuxu Wang, Xiude Chen, Ling Li, Xiling Fu, Dongsheng Gao

**Affiliations:** 1 College of Horticulture Science and Engineering, Shandong Agricultural University, Tai’an, Shandong, China; 2 State Key Laboratory of Crop Biology, Shandong Agricultural University, Tai’an, Shandong, China; 3 Shandong Collaborative Innovation Center for Fruit & Vegetable Production with High Quality and Efficiency, Tai’an, Shandong, China; 4 Laiyang City Bureau of Natural Resources and Planning, Yantai, Shangdong, China; 5 Royal Holloway, University of London, UK

**Keywords:** Bud break, cell cycle, cell wall modification, hormone, peach (Prunus persica), PpEBB1, PpEXBL1

## Abstract

In a previous study we identified EARLY BUD BREAK 1 (EBB1), an ERF transcription factor, in peach (*Prunus persica* var. *nectarina* cultivar Zhongyou 4); however, little is known of how PpEBB1 may regulate bud break. To verify the function of PpEBB1 in bud break, *PpEBB1* was transiently transformed into peach buds, resulting in early bud break. Bud break occurred earlier in *PpEBB1-oe* poplar (*Populus trichocarpa*) obtained by heterologous transformation than in wild type (WT), consistent with the peach bud results, indicating that PpEBB1 can promote bud break. To explore how PpEBB1 affects bud break, differentially expressed genes (DEGs) between WT and *PpEBB1-oe* poplar plants were identified by RNA-sequencing. The expression of DEGs associated with hormone metabolism, cell cycle, and cell wall modifications changed substantially according to qRT-PCR. Auxin, ABA, and total *trans*-zeatin-type cytokinin levels were higher in the *PpEBB1-oe* plants than in WT plants, while the total *N*^6^-(Δ ^2^-isopentenyl)-adenine-type cytokinins was lower. Yeast two-hybrid and bimolecular fluorescence complementation assays verified that a cell wall modification-related protein (PpEXBL1) interacted with PpEBB1 suggesting that PpEBB1 could interact with these cell wall modification proteins directly. Overall, our study proposed a multifaceted explanation for how PpEBB1 regulates bud break and showed that PpEBB1 promotes bud break by regulating hormone metabolism, the cell cycle, and cell wall modifications.

## Teaser

The transcription factor EBB1 plays important roles in regulating bud break in peach trees by regulating hormone metabolism, the cell cycle, and cell wall modifications.

## Introduction

Dormancy is an important physiological phenomenon that allows trees to endure harsh environmental conditions during winter (Chen *et al*., 2016), during which time visible growth of any plant structure is lacking. There are three types of dormancy: paradormancy, endodormancy, and ecodormancy (Lang, 1987). In paradormancy, the growth of lateral buds is suppressed by actively growing parts of the plant, such as the apical meristem; this process is known as apical dominance. Buds in endodormancy without meeting chilling requirements will not grow outward, even under favorable conditions. Following the endodormancy stage, buds become ecodormant and resume growth when conditions become favorable (Shim *et al*., 2014; Castède *et al*., 2015). The transition from active growth to dormancy in the fall is usually induced by short-day conditions or cold temperature; the transition of peach and poplar is induced by short-day conditions. Bud dormancy is an important adaptive mechanism that can protect plants from frost damage either in late spring around the time of bud break or in early fall around the time of growth cessation (Yordanov *et al*., 2014).

Numerous studies have shown that plant hormones control dormancy and bud break, especially auxin, abscisic acid (ABA), gibberellin (GA), and cytokinin (Horvath *et al*., 2008; Balla *et al*., 2011; Aksenova *et al*., 2013; Gillespie and Volaire, 2017). Auxin is considered to play important roles in paradormancy. The classic model for the function of auxin in bud outgrowth involves the transport of auxin from the apical and lateral parts of the stem inhibiting auxin export from axillary buds, suppressing axillary bud outgrowth (Balla *et al*., 2011). However, there is a recent view that auxin export from axillary buds is independent of initial bud growth but is important for sustained bud outgrowth (Barbier *et al*., 2019). This view is also supported by research on pea (*Pisum sativum*) in which the application of inhibitors of both auxin transport and perception had no effects on decapitation-induced early bud outgrowth; instead, the inhibitory effects were apparent after 2 d (Chabikwa *et al*., 2019). ABA is recognized as the key internal factor for entering dormancy and is involved in dormancy induction and maintenance (Horvath *et al*., 2008; Gillespie and Volaire, 2017). The concentration of ABA decreases while that of auxin, cytokinin, and GA increases during dormancy release in herbaceous perennial species (Gillespie and Volaire, 2017). The role of GA and cytokinin application in promoting dormancy release and bud break has been widely studied (Roman *et al*., 2016; Gillespie and Volaire, 2017). Exogenous GA has long been used to break dormancy and promote bud burst, but the role of exogenous GA in dormancy release varies with different species (Gillespie and Volaire, 2017). Cytokinin acts as a primary regulator of dormancy release. Inactivation of cytokinin results in prolonged dormancy, while increased cytokinin activity leads to shortened dormancy (Aksenova *et al*., 2013). Cytokinin treatment can up-regulate the expression of GA biosynthesis genes and down-regulate the expression of GA degradation-related genes significantly (Ni *et al*., 2017). Therefore, crosstalk among different hormones may also play important roles in dormancy regulation. Auxin, cytokinin, and their interaction can regulate the outgrowth of axillary buds suppressed by the shoot tip (Ferguson and Beveridge, 2009). In *Chrysanthemum*, decreased auxin and increased cytokinin coincide with more branches, whereas high auxin levels and decreased cytokinin levels are associated with strong apical dominance (Dierck *et al*., 2016). In recent years, strigolactone was discovered to be a branching inhibitor, and could regulate bud outgrowth by controlling auxin export from axillary buds together with cytokinin (Crawford *et al*., 2010; Shinohara *et al*., 2013; Rameau *et al*., 2014; Waldie and Leyser, 2018). However, to date, the role of all these hormones during the control of growth cessation, bud dormancy, and outgrowth is still not fully understood (Maurya and Bhalerao, 2017).

When buds begin to grow outward, an increase in cell division often occurs (Horvath *et al*., 2003). The plant cell cycle consists of four main stages: DNA synthesis (S), mitosis (M), and two intervening gap phases (G1 and G2) (Velappan *et al*., 2017). Most cells in the buds are arrested at the G1 phase of the cell cycle during dormancy (Devitt and Stafstrom, 1995). Different types of cyclins (CYCs) operate in concert with cyclin-dependent kinases (CDKs) during different cell cycle stages. A-type CDKs (CDKAs) function mainly in the G1–S and G2–M phase transitions, while B-type CDKs (CDKBs) function in the G2–M phase transition. CDKAs form a complex with CYCDs during the G1–S transition, while CDKAs form a complex with CYCAs, promoting the transition to G2. CYCA/B operates together with CDKA and CDKB to regulate the G2–M transition (Velappan *et al*., 2017). The expression of genes related to the G1-to-S-phase transition, such as *D-cyclins* (*CYCD*s) and *CDK*s, is one of the conditions for bud outgrowth (Shimizu-Sato and Mori, 2001; Aksenova *et al*., 2013). A large number of studies have directly linked cell development to signals such as hormones that regulate adventitious bud or axillary bud dormancy (Horvath *et al*., 2003). There is a negative correlation between ABA content and transcript levels of cell cycle genes in grapevine latent buds and shoot apexes. The expression of cell cycle-related genes (*CDKB1/2*, *CYCA1/2/3*, *CYCB*, *CYCD3.2a*) is repressed by ABA, and the expression of cell cycle-related genes is decreased when the ABA content increases during endodormancy (Vergara *et al*., 2017). GA and cytokinin treatment can regulate the expression of genes related to the cell cycle, indicating that these hormones may promote bud outgrowth by regulating the cell cycle machinery in axillary buds, which was shown in *Jatropha curcas* (Ni *et al*., 2017).

Plant cell walls are important for plant growth and development, for protection against cell wall damage detection, and for bioconversion (De Lorenzo *et al*., 2019). Recently, studies have shown that cell walls can regulate seed germination (Bassel, 2016) and bud outgrowth in both annual and perennial plants (Lee *et al*., 2017). Cell walls are composed mainly of cellulose, hemicellulose, pectin, lignin, and proteins (Sarkar *et al*., 2009). Enzymes mediating these components, such as endotransglycosylases/hydrolases (XTHs), pectin methylesterases (PMEs), and expansins, are important for cell wall loosening and for regulating germination. In Arabidopsis seeds, the activity of cell wall enzymes plays an important role in germination, which can lead to radicle protrusion by enabling embryo cell expansion (Sechet *et al*., 2016). The genes related to the promotion of cell expansion are not expressed in dormant seeds (Cadman *et al*., 2006). In Norway spruce, the permeability of the cell wall in growing shoot tips can be affected by the photoperiod, thereby regulating bud growth–dormancy cycling by influencing the transport of water into the buds (Lee *et al*., 2017).

Bud dormancy has an important impact on fruit production, especially in perennial deciduous fruit trees (Sun *et al*., 2016). However, the key genes that function in the transition between dormancy and regrowth are still poorly understood. *DORMANCY ASSOCIATED MADS-BOX* (*DAM*) genes were initially thought to be associated with dormancy because *evergrowing* peach mutants, which have a deletion in a series of *DAM* genes, do not stop growing or develop buds under short-day conditions (Bielenberg *et al*., 2004, 2008). *DAM* genes have been identified as being critical factors controlling endodormancy and dormancy release in many species. For example, *DAM5* and *DAM6* in peach (*Prunus persica*) may function in the chilling requirement of lateral buds and may be negatively correlated with bud burst (Yamane *et al*., 2011). The transcript levels of *DAM1* and *DAM3* in pear (*Pyrus pyrifolia*) peak at the endodormancy stage but then decrease at the ecodormancy stage (Niu *et al*., 2016). A study on *Prunus mume* indicated that PmDAMs interact with other PmDAMs or themselves to form protein complexes (Zhao *et al*., 2018*a*). Moreover, in pear, the expression of DAM can be directly promoted by the accumulation of C-repeat binding factor (CBF) and then inhibits *FLOWERING LOCUS T* (*FT*) expression to induce endodormancy (Niu *et al*., 2016). Recently, EARLY BUD BREAK 1 (EBB1) was identified as promoting bud break in poplar by reactivating meristematic activity after winter dormancy (Yordanov *et al*., 2014), and homologs of EBB1 in apple, pear, grape, and spruce may function in bud break (Busov *et al*., 2016; Wisniewski *et al*., 2015; Anh Tuan *et al*., 2016). However, it remains unclear how EBB1 regulates bud break. Here, the EBB1 in peach, which was identified in our previous study based on its expression level during the dormancy phase (Zhao *et al*., 2018*b*), was heterologously transformed into poplar because there were no transformation methods in peach, and RNA-sequencing (RNA-seq) was used to investigate the mechanism by which PpEBB1 regulates bud break.

## Materials and methods

### Plant materials

Samples of buds of peach (*Prunus persica* var. *nectarina*) cultivar Zhongyou 4 were harvested from 5-year-old trees growing at the Horticulture Experimental Station of Shandong Agricultural University, Tai’an, China. All trees were grown in accordance with standard agricultural practices. Leaf bud samples were collected from first-year branches approximately every 2 weeks from October 2016 until April 2017. The samples were immediately flash frozen in liquid nitrogen and subsequently stored at −80 °C for total RNA extraction and qRT-PCR-based experiments.

### Construction of viral vectors and transient expression in buds

Transient expression assays were performed in peach buds. The coding DNA sequence (CDS) of *PpEBB1* was cloned and inserted into an IL60 virus plasmid vector. The shoots used for transient expression were randomly collected and cultivated under the following conditions: 16 h of light at 25 °C with an artificial fluorescence lamp (200 µmol m^−2^ s^−1^) and 8 h of darkness at 18 °C. The relative humidity was maintained at 70%. Approximately 2 μg ml^−1^ of the IL60 and PpEBB1-IL60 plasmids was introduced into peach buds as the control and treatment condition, respectively.

### Identification and phylogenetic analysis of *PpEBB1*

The most current nectarine protein and CDSs were downloaded from the JGI database (https://phytozome.jgi.doe.gov/pz/portal.html#!info?alias=Org_Pprsica). To study the phylogenetic relationship between *PpEBB1* and homologous genes from other plant species, a phylogenetic tree was created via MEGA 5.05 software, and the full-length protein sequences were subjected to multiple alignment by the neighbor-joining method of ClustalW; the bootstrap test was replicated 1000 times.

### Total RNA extraction and quantitative real-time PCR-based experiments

Total RNA was isolated via an RNAprep Pure Plant Kit (polysaccharide- and polyphenolic-rich) (Tiangen, Beijing, China). The quality and quantity of the RNA were determined via 1.0% agarose gel electrophoresis and a NanoPhotometer P360 (Implen, Munich, Germany). Reverse transcription was performed with a PrimeScript RT Reagent Kit and gDNA Eraser (Perfect Real Time) (Takara Biotechnology, Dalian, China). The primers used for qRT-PCR are listed in Supplementary Table S1 at *JXB* online. SYBR Premix Ex Taq (Takara Biotechnology) was used to perform quantitative real-time PCR (qRT-PCR). The thermal cycling conditions for qRT-PCR were as follows: predenaturation at 95 °C for 10 min followed by 40 cycles of denaturation at 95 °C for 5 s, annealing at 60 °C for 30 s, and extension. Each sample was replicated three times.

### Vector construction and overexpression of *PpEBB1* in poplar

The *PpEBB1* gene was obtained via a pair of primers (forward, 5′-ATGGAAGAGGCATTTAGAAGG-3′ and reverse, 5′-GTTCTGGACCCTGGC-3′) with the additional restriction enzyme cutting sites of *Sal*I and *Bam*HI to allow for the direct cloning of the *PpEBB1* gene into the pRI101 vector under the control of the CaMV35S promoter. The resulting *35S:PpEBB1* fusion construct was subsequently introduced into *Agrobacterium tumefaciens* strain GV3101.

Poplar (*Populus trichocarpa*) was used for heterologous transformation. Poplar seedlings were propagated by stem segments in aseptic glass bottles containing Murashige and Skoog (MS) media supplemented with 0.1 mg l^−1^ naphthylacetic acid (NAA). The poplar seedlings were subsequently grown at temperatures of 21–25 °C/15–18 °C (day/night) under a 12 h light (200 µmol m^−2^ s^−1^)/12 h dark photoperiod. Approximately 1 month later, approximately the fourth to sixth expanded leaves from the apex were used for injection. The leaves were cultivated on MS medium supplemented with 0.1 mg l^−1^ NAA, 0.2 mg l^−1^ 6-benzylaminopurine, 0.01 mg l^−1^ thidiazuron, 800 mg l^−1^ timentin, and 50 mg l^−1^ kanamycin after injection. One or two months later, the small seedlings growing from the leaves were transferred to MS medium supplemented with 0.1 mg l^−1^ NAA and 50 mg l^−1^ kanamycin to grow roots. The screened transgenic lines were then transplanted into soil in a greenhouse.

### RNA-sequencing data analysis

*Populus trichocarpa* genome annotations were downloaded from the JGI database (http://genome.jgi.doe.gov/Ptrichocarpa/download/_JAMO/587b0ae47ded5e4229d885bd/Ptrichocarpa_444_v3.1.gene_exons.gff3.gz?requestTime=1501142324). All reads were of high quality according to the distribution of the raw data. To identify differentially expressed genes (DEGs), DESeq2 v1.6.3 was used. Transcript abundance was normalized to fragments per kilobase of transcript per million mapped reads (FPKM) (Trapnell *et al*., 2010). The *P*-value and fold change were used to screen the DEGs, and genes with |log2 ratio|≥1 and *q*≤0.05 were identified as DEGs. DEGs were implemented by the hypergeometric test for Gene Ontology (GO, http://geneontology.org/) enrichment analysis. GO terms with *q*<0.05 were considered to be significantly enriched. The Kyoto Encyclopedia of Genes and Genomes (KEGG) enrichment of DEGs was implemented by a hypergeometric test, in which the *P*-value was adjusted by multiple comparisons, yielding a *q*-value. KEGG pathways with *q*<0.05 were considered significantly enriched.

### Yeast two-hybrid assay

A yeast two-hybrid (Y2H) library was constructed with peach buds. PpEBB1^1−424 aa^, PpEBB1^1−120 aa^, and PpEBB1^121−424 aa^ were cloned and inserted into a pGBKT7 vector as bait. The vectors were transformed into Y2H-competent cells and cultured at 30 °C on −Trp, −Trp/X-α-gal, and −Trp/X-α-gal/aureobasidin A (AbA) media. PpEXLB was cloned and inserted into a pGADT7 vector as prey. The bait and prey were subsequently transformed into Y2H-competent cells and cultured on −Trp/−Leu, −Leu/−Trp/−His/−Ade, and −Leu/−Trp/−His/−Ade media with X-α-gal.

### Bimolecular fluorescence complementation assay

The CDSs of *PpEBB1* and *PpEXLB* were transferred into pSPYNE and pSPYCE, respectively. The recombinant plasmids were then transformed into *A. tumefaciens* GV3101. The PpEBB1-pSPYNE and PpEXLB-pSPYCE plasmids were mixed together (1:1), after which onion epidermal cells were transfected with the mixture for 30 min; a mixture of PpEBB1-pSPYNE and pSPYCE was used as a control. The onion epidermal cells were then transferred to MS medium. Forty-eight hours later, the onion epidermal cells were observed at an excitation wavelength of 514 nm under a laser scanning confocal microscope (LSM880) (Carl Zeiss, Oberkochen, Germany).

### Hormone determination

Samples (approximately 0.5 g of fresh weight per sample) were taken from *PpEBB1-oe* plants and wild-type (WT) and immersed immediately in liquid nitrogen. The contents of endogenous plant hormones were determined according to the method of Cai *et al*. (2015). The experiment was repeated at least three times for each sample.

### Statistical analysis

Charts were constructed with GraphPad Prism software. Statistical analysis was performed with IBM SPSS Statistics 19 software, and Duncan’s test was used to analyse the data. A significance level of *P*<0.05 was applied.

## Results

### *PpEBB1* may participate in bud break

**Fig. 1.**
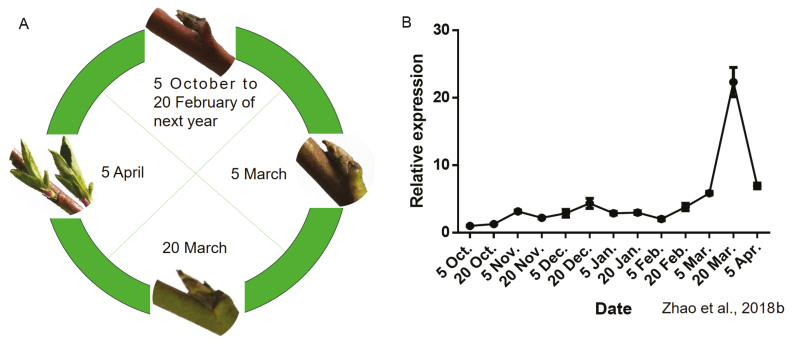
Morphology of peach buds and the relative expression of *PpEBB1*. (A) Morphology of peach buds during the various stages of dormancy and bud break from 5 October 2016 to 5 April 2017. (B) Expression level of *PpEBB1* during the various stages of dormancy and bud break from 5 October 2016 to 5 April 2017. The values represent the means ±SD of three replicates.

Peach buds were wrapped in thick bud scales and displayed no visible growth from 5 October 2016 to 20 February 2017. On 5 March, bud growth was obvious. The buds began to break away from the bud scales on 20 March, and new leaves grew outward on 5 April (Fig. 1A). In our previous study, we identified a gene named *PpEBB1* that might function in the bud break of peach. The relative expression of *PpEBB1* was very low and nearly constant during dormancy, whereas it began to be up-regulated on 20 February, peaked on 20 March, then decreased rapidly on 5 April, consistent with the date of bud break (Fig. 1B) (Zhao *et al*., 2018*b*).

### *PpEBB1* can promote bud break

**Fig. 2.**
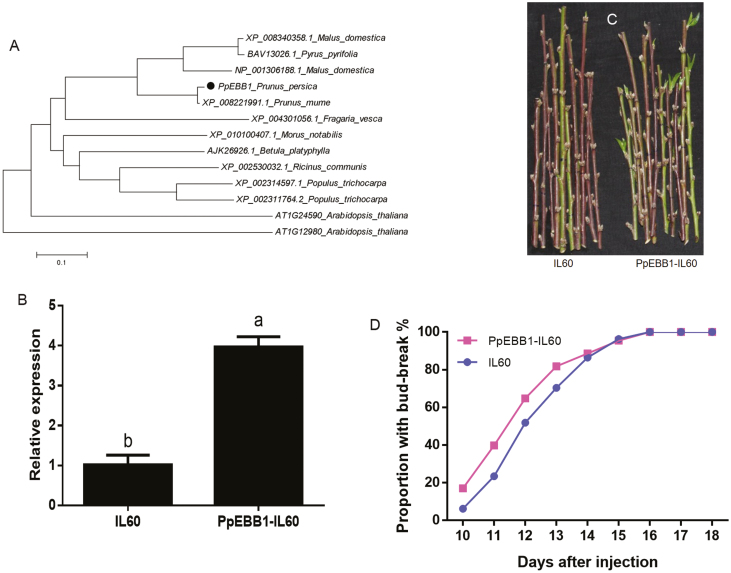
Transient expression of *PpEBB1* in peach buds. (A) Unrooted neighbor-joining tree of orthologous genes of *PpEBB1* from different species. (B) Transient conversion of *PpEBB1* in peach buds with an *EBB1* overexpression level three times greater than that in the control. The values represent the means ±SD of three replicates, and the different letters above the bars represent signiﬁcant differences; *P*<0.05. (C, D) Comparison of peach buds injected with a PpEBB1-IL60 vector and an empty IL60 vector (as a control). Ninety buds were divided into three groups as three replicates for transformation in each treatment. The proportion of bud break was calculated according to the sum of the buds in the three groups, and the buds that ultimately burst were included in the total buds.

Phylogenetic analysis revealed that *PpEBB1* was highly homologous to *EBB1* in apple and pear (Fig. 2A), which may function in bud break because of its up-regulated expression when buds began to regrow. To elucidate the function of *PpEBB1* in bud break, the *PpEBB1* gene was cloned, and a PpEBB1-IL60 construct was generated; the construct was transformed into dormant peach buds, and an empty IL60 vector was used as a control. The transcript level of *PpEBB1* significantly increased in PpEBB1-IL60-transfected buds compared with control buds (Fig. 2B), and the date of bud break was earlier for the buds transfected with *PpEBB1* than for the buds transfected with the control (Fig. 2C, D).

**Fig. 3.**
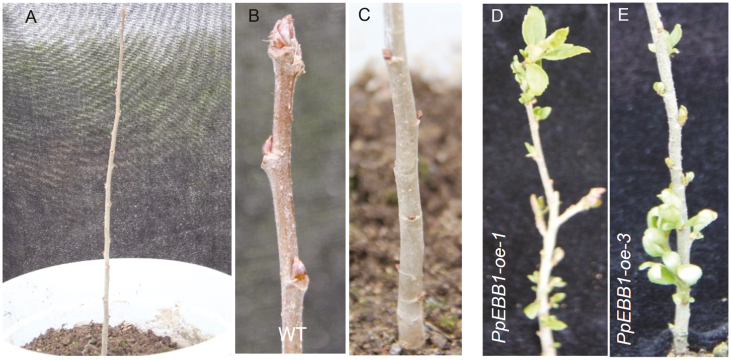
Heterologous transformation of *PpEBB1* induces early bud burst in poplar. Transgenic plants were planted in a greenhouse. Images were taken on 8 March 2019. (A–C) are WT plants, (D, E) are transgenic plants.

Moreover, transgenic poplar plants overexpressing *PpEBB1* (*PpEBB1-oe*) (Supplementary Fig. S1) were screened and planted in the greenhouse. The *PpEBB1-oe* plants (Fig. 3D, E) reactivated growth in early spring on 8 March when the WT plants remained dormant (Fig. 3A–C). Thus, regrowth occurred earlier in the transgenic *PpEBB1-oe* plants than in the WT plants. These results indicated that *PpEBB1* plays important roles in promoting bud break.

### GO and KEGG analyses

To explore how *PpEBB1* acts on bud break, RNA-seq was used to study the transcriptomic changes in the *EBB1-oe* poplar plants. A total of 2534 DEGs were identified (Supplementary Dataset S1), including 1525 genes whose expression was up-regulated and 1009 genes whose expression was down-regulated in the *PpEBB1-oe* plants compared with the WT plants. The transcriptomic data were successfully validated by qRT-PCR for a subset of 15 DEGs (Supplementary Fig. S2). DEGs were subjected to KEGG analysis, which highlighted several pathways, including the plant hormone signal transduction, zeatin biosynthesis, and tryptophan metabolism pathways (Supplementary Dataset S2). DEGs were also subjected to GO analysis and were classified into three categories: biological processes, cellular components, and molecular functions (data not shown). We mainly focused on GO terms associated with biological processes (Supplementary Dataset S3). From these GO terms, genes involved in hormone metabolism, especially that of auxin and cytokinin, the cell cycle process, and cell wall modification, which are processes that also function in dormancy, were significantly enriched; therefore, these genes were selected for further analysis.

### DEGs related to cytokinin and auxin metabolism with the overexpression of *PpEBB1*

**Fig. 4.**
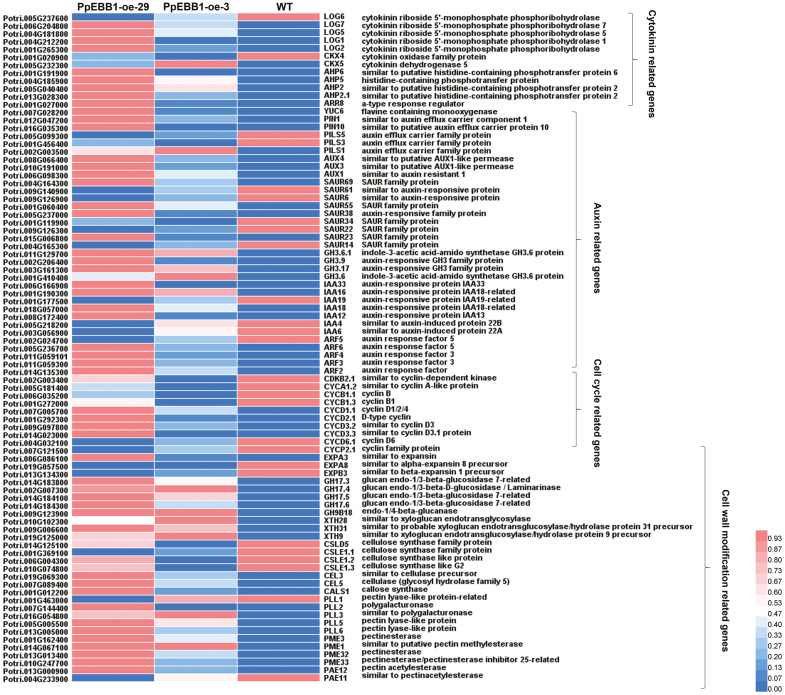
Heat map of the DEGs between WT and transgenic (*PpEBB1*-oe-3 and *PpEBB1*-oe-29) poplar plants mainly included cytokinin and auxin metabolism, cell cycle, and cell wall modification-related genes. The annotation of each gene was retrieved from https://phytozome.jgi.doe.gov/pz/portal.html#, and the abbreviated name is provided for reference. The qRT-PCR data for the heat map are the means of three replicates.

Many DEGs associated with auxin and cytokinin metabolism, the cell cycle process and cell wall modification were selected from the transcriptomic data, and the relative expression of these genes in *EBB1-oe* plants and WT plants was assessed by qRT-PCR (Supplementary Dataset S4). A heat map (Fig. 4) was formed from the qRT-PCR data. Many genes associated with cytokinin and auxin metabolism showed marked changes in expression in the comparison of *EBB1-oe* and WT plants (Fig. 4; Supplementary Dataset S4). Among the DEGs associated with cytokinin biosynthesis, which were mainly lonely guy (*LOG*) and dehydrogenase-like cytokinin oxidase/dehydrogenase (*CKX*) genes, the expression levels of most were up-regulated in the *EBB1*-*oe* plants, except for *LOG6* and *CKX4*. Regarding the cytokinin signaling component, there were four Arabidopsis histidine phosphotransfer proteins (*AHP*s) among the DEGs, and the expression level of all of these genes was higher in the transgenic plants than in the WT plants. The expression of *ARR8*, a type-A Arabidopsis response regulator, was up-regulated in the transgenic plants in our study. These results indicated that *PpEBB1* could regulate cytokinins through the cytokinin biosynthesis pathway and signaling response.

For the auxin metabolism pathway, an auxin biosynthesis gene, *YUCCA 6* (*YUC6*), was up-regulated in the *EBB1*-*oe* plants. The expression levels of PIN-formed genes (*PIN*s) and auxin-resistant mutation genes (*AUX*s), which encode auxin efflux and influx carrier proteins, also changed. In addition, genes involved in auxin signaling, including members of the small auxin-up RNA (*SAUR*), *GH3* and auxin/indole-3-acetic acid (*Aux/IAA*) family, were identified, and nine, four, and seven genes in the *SAUR*, *GH3*, and *Aux/IAA* families, respectively, were differentially expressed. The expression levels of four and one auxin response factor genes (*ARF*s) were up-regulated and down-regulated, respectively. These results indicated that *PpEBB1* can regulate auxin through auxin biosynthesis, transport, and signaling responses.

**Fig. 5.**
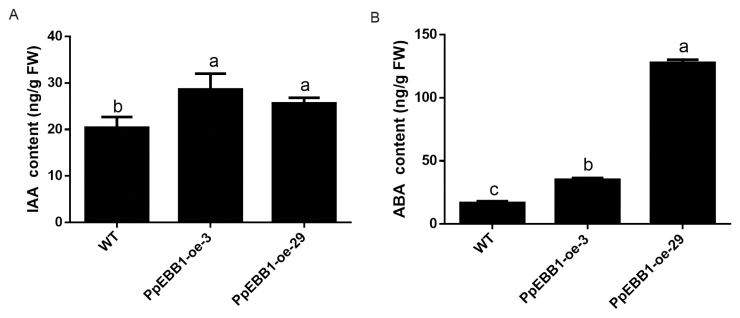
Hormone content in the WT and transgenic plants. (A) Content of IAA; (B) content of ABA. The values represent the means ±SD of three replicates, and the different letters above the bars represent signiﬁcant differences; *P*<0.05.

Owing to the important effect of *PpEBB1* on cytokinin- and auxin-related genes, the levels of indoleacetic acid (IAA) and cytokinins were measured. IAA significantly increased in the transgenic plants compared with the WT plants (Fig. 5A). Six types of cytokinins were detected, and only the *trans*-zeatin (tZ) content was higher in the *PpEBB1-oe* plants than in the WT plants. The total tZ-type cytokinin was higher in the *PpEBB1-oe* plants than in the WT plants, while the total *N*^6^-(Δ ^2^-isopentenyl)-adenine (iP)-type cytokinin was lower (Table 1). In addition to cytokinin and auxin, some genes related to GA and ABA were also differentially expressed according to the transcriptomic data. Owing to the important role of these hormones during dormancy and bud break, the content of these hormones was also determined. The results showed that the ABA content increased in the *PpEBB1-oe* plants (Fig. 5B), which was unexpected. Three types of GA were detected: GA1 and GA4 were detected only in the WT plants, and GA3 was detected only in the *PpEBB1-oe* plants (Table 2).

**Table 1.** Levels of different types of cytokinin in *PpEBB1-oe* plants

Sample	iP	iPR	tZ	tZR	cZ	cZR	Total tZ-types	Total iP-types
WT	0.09±0.01	21.34±0.25	0.25±0.02	0.22±0.01	0.27±0.02	17.33±0.73	0.47±0.03	21.43±0.25
PpEBB1-oe-3	0.05±0.00	5.26±0.04	0.36±0.04	0.43±0.02	0.30±0.03	4.77±0.24	0.79±0.06	5.31±0.04
PpEBB1-oe-29	0.04±0.00	12.00±0.42	0.13±0.01	1.32±0.11	0.05±0.01	6.48±0.09	1.44±0.12	12.04±0.42

Values shown are means ±SD (ng g^−1^). cZ, *cis*-zeatin; cZR, cZ riboside; iP, *N*^6^-(Δ ^2^-isopentenyl)-adenine; iPR, iP riboside; tZ, *trans*-zeatin; tZR, tZ riboside.

**Table 2.** Levels of different types of GA in *PpEBB1-oe* plants

Sample	GA1	GA3	GA4
WT	0.67±0.01	n.d.	0.08±0.00
PpEBB1-oe-3	n.d.	0.11±0.01	n.d.
PpEBB1-oe-29	n.d.	0.23±0.01	n.d.

Values shown are means ±SD (ng g^−1^). n.d., not detected.

### DEGs related to the cell cycle and cell wall modification with the overexpression of *PpEBB1*

DEGs related to the cell cycle and cell wall modification were detected (Fig. 4; Supplementary Dataset S4). Many CYC genes were differentially expressed, such as *CYCD1.1*, *CYCD2.1*, and *CYCD3.2/3.3*, whose expression was up-regulated, while the expression levels of *CYCA1.2*, *CYCB1.1/1.3*, *CYCD6.1*, and *CYCP2.1* were down-regulated. The expression of a CDK-encoding gene, *CDKB2*, was down-regulated in the transgenic plants compared with the WT plants. A large number of the DEGs associated with expansins and the metabolism of cell wall components were identified on the basis of the GO annotations. The heat map showed that the expression levels of three expansin, five glycosyl hydrolase, three xyloglucan endotransglucosylase/hydrolase, six cellulose, one callose, and 11 pectin metabolism-related genes were altered (Fig. 4; Supplementary Dataset S4). These results indicated that *PpEBB1* plays a crucial role in the cell cycle and cell wall modification.

### PpEBB1 interacts with PpEXLB1

**Fig. 6.**
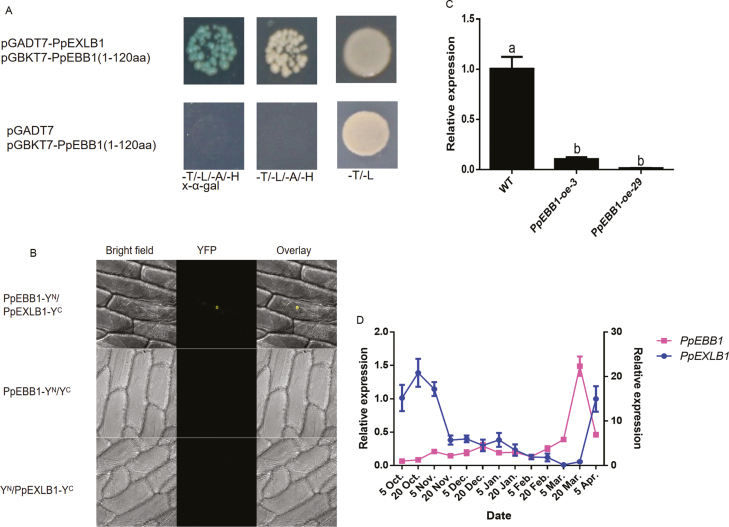
PpEBB1 interacts with PpEXLB, and the expression pattern of PpEXLB during the dormancy stage. (A) Interaction between PpEBB1 and PpEXLB in a Y2H assay. Because of the autoactivation of the full-length PpEBB1 protein, we used aa 1–120 to perform the experiment. (B) Interaction between PpEBB1 and PpEXLB in a BiFC assay. (C) Compared with those in the WT plants, the expression levels of *PpEXLB1* in two lines of *PpEBB1-oe* poplar decreased. (D) Expression level of *PpEXLB1* during different dormancy stages from 5 October 2016 to 5 April 2017; blue represents *PpEXLB1* (left axis), and purple represents *PpEBB1* (right axis). The values represent the means ±SD of three replicates, and the different letters above the bars represent signiﬁcant differences; *P*<0.05.

To examine how *PpEBB1* is involved in bud break and to identify the genes that may interact with *PpEBB1*, a Y2H library was constructed from buds collected from dormancy to bud break. The N-terminus (1–120 amino acids (aa)) of PpEBB1 was used for screening interacting genes whose products contain an AP2/ERF domain, as the full-length protein is autoactivated (Supplementary Fig. S3). Ten genes (Supplementary Table S2) were screened and subsequently tested for their ability to interact with PpEBB1. The full-length cDNA of these genes was inserted into a pGADT7 vector as prey and together with BD-EBB1^1−120aa^ was used for Y2H assays. The *expansin-like B1* (*EXLB1*; *Prupe.5G057900*) gene was found to interact with the N-terminal AP2/ERF domain (1–120 aa) of EBB1 (Fig. 6A), which might function in loosening the cell wall to regulate cell expansion. The interaction between PpEBB1 and PpEXLB was also verified by bimolecular fluorescence complementation (BiFC) assays (Fig. 6B).

In *PpEBB1* transgenic plants, the relative expression of *PpEXLB1* was down-regulated (Fig. 6C). The expression levels of *PpEXLB1* in peach buds were also investigated in parallel with *PpEBB1* from late autumn to early spring. In the growth cessation stage, the expression level of *PpEXLB1* was high, while the level was decreased in the dormancy stage and was lowest on 5 March, the time at which the expression of *PpEBB1* was increasing. The expression of *PpEXLB* was then rapidly up-regulated on 5 April, which was the opposite of the expression pattern of *PpEBB1* (Fig. 6D).

## Discussion

The *EBB1* gene in peach was identified; it has high sequence homology with those of apple, pear, and poplar (Fig. 2A). The relative expression of *EBB1* during the dormancy period in apple (Wisniewski *et al*. 2015) and pear (Anh Tuan *et al*., 2016) was similar to that in peach in our study (Fig. 1), indicating that *EBB1* might be associated with bud break. The results for both transformed peach buds and transgenic poplar (Figs 2C, D, 3) strongly confirmed that *PpEBB1* plays an important role in the resumption of growth after winter dormancy. First, the expression level of *EBB1* in buds was very low during the majority of the dormancy period but sharply increased at bud break, after which it rapidly decreased. Second, the overexpression of *PpEBB1* was sufficient to accelerate bud burst. Third, these results were consistent with those of studies on apple, pear, and poplar (Yordanov *et al*., 2014; Wisniewski *et al*. 2015; Anh Tuan *et al*., 2016). Therefore, the role of *EBB1* in promoting bud break is important and relatively conserved across perennial woody plant species.

The molecular mechanism by which *EBB1* regulates bud break remains unclear; thus, we used RNA-seq to study the transcriptomic changes in *PpEBB1-oe* poplar plants. GO analysis of the DEGs revealed terms associated with many processes critical for bud break, including hormone metabolism, the cell cycle, and cell wall modification (Supplementary Dataset S3), suggesting that *PpEBB1* might promote bud break by regulating the metabolism of hormones, the cell cycle, and cell wall modification.

**Fig. 7.**
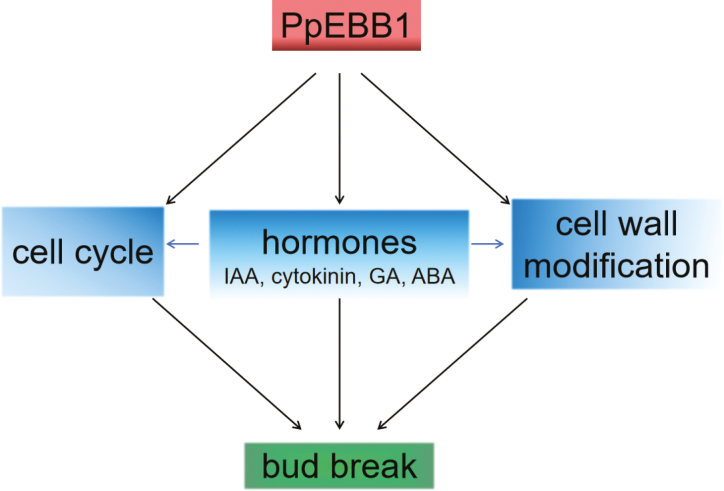
Hypothetical model of PpEBB1 function in bud break. PpEBB1 can regulate genes associated with hormones, the cell cycle and cell wall modifications to promote bud break. In addition to accelerating bud break directly, hormones may act by regulating the cell cycle and cell wall modifications.

Among the DEGs associated with hormones, we focused mainly on genes related to cytokinin and auxin because genes involved in the metabolism of these two hormones were more enriched according to the GO analysis (Supplementary Dataset S3). Cytokinin is known to promote bud break. A previous study (Roman *et al*., 2016) in rose showed that cytokinin was the key factor that controlled bud outgrowth due to the exogenous supply of synthetic cytokinin 6-benzylaminopurine, which led to bud outgrowth in the dark, whereas two inhibitors of cytokinin perception repressed bud outgrowth, demonstrating the significant role of cytokinin in bud outgrowth. In Arabidopsis, cytokinin plays an important role in the formation of the shoot apical meristem (SAM), as the content of cytokinin causes changes in the SAM (Tokunaga *et al*., 2012). Moreover, in the SAM, the expression of *WUSCHEL* (*WUS*) is positively regulated by cytokinin (Gordon *et al*., 2007). Recent studies have shown that type-B ARRs can directly bind to the promoter region of *WUS* to promote its expression (Wybouw and De Rybel, 2019). Among the different types of cytokinin, tZ and iP generally presented relatively high activity, while *cis*-zeatin (cZ) presented relatively low or no activity (Sakakibara, 2006). In our study, overexpression of *PpEBB1* improved the total content of tZ-type cytokinin and reduced the total iP-type cytokinin (Table 1). A study in rose showed that light-induced cytokinin accumulation in buds preferentially induced the tZ-type compared with the iP-type cytokinin (Roman *et al*., 2016). The levels of tZ and iP are balanced in plants by the antagonistic regulation of cytokinin-related enzymes, which interact with other factors, such as auxin and ABA, to regulate plant responses (Sakakibara, 2006). In addition, exogenous application of cytokinin can accelerate bud burst in rose, perhaps by inducing mechanisms closely related to germination, such as the expression of sugar-, IAA- and cell cycle-related genes (Roman *et al*., 2016). Cytokinin was shown to be capable of regulating cell division by interacting with CYCD3 and controlling the G2–M and G1–S transitions in the cell cycle (Zhang *et al*., 2005; Dewitte *et al*., 2007; Schaller *et al*., 2014). These results confirm that the changes in cytokinin levels influenced by *PpEBB1* may act as a factor in promoting bud break or by regulating other bud break-related genes and metabolic pathways indirectly (Fig. 7).

A study (Yordanov *et al*., 2014) of *EBB1* in poplar suggested a possible connection between *EBB1* and auxin signaling because genes involved in auxin signaling were enriched in *EBB1*-modified transgenics, and the orthologous gene *DRN/DRNL* in Arabidopsis can act via the auxin signal transduction pathway (Cole *et al*., 2009). DEGs related to auxin synthesis and transport and auxin signaling pathways were significantly enriched in *PpEBB1-oe* poplar plants in our study. The up-regulated expression of the auxin synthesis-related gene *YUC6* was strongly correlated with an increased IAA content in the transgenic plants in this study (Figs 4, 5A; Supplementary Dataset S4). Although it is well known that auxin primarily plays a significant role in paradormancy (Balla *et al*., 2011), the function of auxin in bud break is not clear. By using qRT-PCR, we found that the expression levels of a large number of genes associated with auxin signaling pathways were altered (Fig. 4; Supplementary Dataset S4), indicating that *EBB1* plays a very important role in auxin signaling. The auxin efflux carrier PIN3 was important for axillary bud outgrowth and could be directly repressed by BRC1 in cucumber (*Cucumis sativus* L.) (Shen *et al*., 2019). In our study, the expression of two *PIN* genes was up-regulated in the *EBB1-oe* plants, suggesting that *EBB1* may regulate the transport of auxin. PIN protein polarization at the plasma membrane could also be promoted by cytokinin in Arabidopsis, resulting in bud outgrowth due to auxin export from buds (Waldie and Leyser, 2018). ABA is widely considered to be involved in dormancy induction and bud outgrowth inhibition, while GA breaks dormancy and promotes bud break. However, in our experiment, the *EBB1-oe* poplar plants, which displayed early bud break, presented increased ABA levels, and the content of GA1, GA3, and GA4 was completely different between the *PpEBB1-oe* plants and the WT plants (Table 2), indicating that the levels of a particular hormone, such as high levels of ABA, have a limited effect on dormancy and bud break. Sechet *et al*. (2016) also reported that early germination is not always accompanied by low ABA and high GA contents in Arabidopsis seeds. These results indicate that bud break may require a balance of various hormones rather than depending on one hormone; this is currently unclear.

Vegetative bud growth following dormancy release is often accompanied by increased cell division. *DRN/DRNL* affects cell cycle progression and provides local competence for the G1–S transition in the SAM of Arabidopsis (Seeliger *et al*., 2016). DEGs associated with the cell cycle, mainly CYCs and CDKs, were identified in our study (Fig. 4; Supplementary Dataset S4). These results confirmed that *EBB1* has a significant influence on cell division by regulating the expression of cell cycle-related genes, which may be one of the reasons *EBB1* promotes bud break. A study (Anh Tuan *et al*., 2016) of EBB in pear reported that EBB could activate the expression of *CYCD3* genes by directly binding to their promoter or by interacting with other transcription factors first, after which the resulting complex increased the activities of the *CYCD3* promoters, or the activity of the *CYCD3* promoter could be enhanced by an intermediate transcription factor, which could be up-regulated by EBB. In addition, the cell cycle could be regulated directly by plant hormones. Auxin stimulates the G1–S transition of the cell cycle to promote lateral root initiation, and cytokinin interacts with CYCD3 in shoot tissue (Zhang *et al*., 2005; Dewitte *et al*., 2007); CYCB and CDKB could be regulated by hormones such as auxin, cytokinin and GA (Francis and Sorrell, 2001). Hormones including ABA, GA, auxin, and cytokinin have distinct functions in the regulation of the cell cycle (Francis and Sorrell, 2001). Therefore, EBB1 could regulate bud break by directly activating cell cycle regulatory genes or by indirectly mediating cell cycle-related genes via hormones (Fig. 7), facilitating the reinitiation of cell division and inducing bud break.

Research on the role of the cell wall in the bud break of perennial woody plants is rare. Microarray analyses showed that cell wall remodeling processes play important roles in germination in Arabidopsis (Penfield *et al*., 2006; Morris *et al*., 2011; Endo *et al*., 2012; Dekkers *et al*., 2013). The expression levels of genes associated with cell wall modification, including those involved in the metabolism of pectin, cellulose, hemicellulose, glycosyl, callose, and expansins, changed significantly when *EBB1* was overexpressed in our study (Fig. 4; Supplementary Dataset S4). Cell wall modifications, such as pectin modification and activity of expansins and XTH, have been proposed to regulate the seed-to-seedling transition (Bassel, 2016). Loss of function of AtXTH31/XTR8 promotes germination in Arabidopsis (Endo *et al*., 2012). Sechet *et al*. (2016) also reported that *xyl1* mutants showed reduced dormancy and could germinate on media lacking gibberellin. Pectin demethyl-esterification regulated by PME is thought to regulate cell expansion (Bordenave and Goldberg, 1994). Pectin demethyl-esterification and callose were found to be associated with bud development and played a role in growth–dormancy cycling by influencing cell wall permeability in Norway spruce (Lee *et al*., 2017). These results demonstrated that the cell wall might also be important for the bud break of perennial woody plants, confirming that cell wall modification might be one of the ways EBB1 influences bud break. Our Y2H and BiFC assay investigating the interaction between PpEBB1 and PpEXLB (Fig. 6A, B) further clarified that PpEBB1 could regulate cell wall remodeling by directly interacting with cell wall modification-related genes. A study on rose (Roman *et al*., 2016) showed that the expression of *RhEXP* increased during bud outgrowth and that the activation of the organogenic activity of SAM in response to cytokinin appeared to be mediated by RhEXP. In addition, cell wall modification could also be regulated by hormones. Previous research showed that activity of a pectate lyase could be induced by auxin in *Zinnia elegans* (Domingo *et al*., 1998). GO enrichment analysis of excess and deficient levels of cytokinin revealed several cytokinin-related genes involved in cell wall modification (Maruyama-Nakashita *et al*., 2004; Hawkesford and Kok, 2006; Takahashi *et al*., 2011; Skalák *et al*., 2019). Recently, a study investigating the interplay between cytokinin and expansins in Arabidopsis roots revealed that EXPA1 could be activated by ARR1 (Pacifici *et al*., 2018). On the basis of our results, EBB1 directly regulated cell wall modification by interacting with related proteins, such as PpEXLB, or indirectly regulated this process by first mediating hormone levels (Fig. 7).

## Conclusion

The *EBB1* gene is involved in the acceleration of bud break, and this function is conserved in perennial woody plants. Understanding the mechanism by which EBB1 functions in bud break can provide a new way for dormancy-related molecular breeding and genetic engineering to be used to develop plants that can avoid bud damage. The results of our study suggest that EBB1 promotes bud break by regulating various mechanisms, such as hormones, cell division, and cell wall modification, and these factors operate synergistically to promote bud break (Fig. 7).

## Supplementary data

Supplementary data are available at *JXB* online.

Dateset S1. DEGs in EBB1-oe lines compared with WT.

Dateset S2. KEGG pathways identified by differentially expressed genes in EBB1-oe poplar.

Dateset S3. Ontology classification associated with BP of differentially expressed genes in EBB1-oe poplar.

Dateset S4. Relative expression of genes are associated with auxin and cytokinin metabolism, cell cycle, and cell wall modification (data presented are the means of three replicates).

Fig. S1. Identification of transgenic poplar lines.

Fig. S2. Verification of RNA-seq results via qRT-PCR.

Fig. S3. Autoactivation of the full-length PpEBB1, PpEBB1^1−120aa^, and PpEBB1^120−424aa^ sequences by a Y2H assay.

Table S1. Primers used for qRT-PCR in this study.

Table S2. Genes that may interact with PpEBB1, as screened by Y2H.

eraa119_suppl_Supplementary_Material

eraa119_suppl_Supplementary_Data_S1

eraa119_suppl_Supplementary_Data_S2

eraa119_suppl_Supplementary_Data_S3

eraa119_suppl_Supplementary_Data_S4

## References

[CIT0001] Aksenova NP, Sergeeva LI, Konstantinova TN, Golyanovskaya SA, Kolachevskaya OO, Romanov GA 2013 Regulation of potato tuber dormancy and sprouting. Russian Journal of Plant Physiology 60, 301–312.

[CIT0002] Anh Tuan P, Bai S, Saito T, Imai T, Ito A, Moriguchi T 2016 Involvement of *EARLY BUD-BREAK*, an AP2/ERF transcription factor gene, in bud break in Japanese pear (*Pyrus pyrifolia* Nakai) lateral flower buds: expression, histone modifications and possible target genes. Plant & Cell Physiology 57, 1038–1047. 26940832 10.1093/pcp/pcw041

[CIT0003] Balla J, Kalousek P, Reinöhl V, Friml J, Procházka S 2011 Competitive canalization of PIN-dependent auxin flow from axillary buds controls pea bud outgrowth. The Plant Journal 65, 571–577. 21219506 10.1111/j.1365-313X.2010.04443.x

[CIT0004] Barbier FF, Dun EA, Kerr SC, Chabikwa TG, Beveridge CA 2019 An update on the signals controlling shoot branching. Trends in Plant Science 24, 220–236. 30797425 10.1016/j.tplants.2018.12.001

[CIT0005] Bassel GW 2016 To grow or not to grow? Trends in Plant Science 21, 498–505. 26934952 10.1016/j.tplants.2016.02.001

[CIT0006] Bielenberg DG, Wang Y, Fan S, Reighard GL, Scorza R, Abbott AG 2004 A deletion affecting several gene candidates is present in the *Evergrowing* peach mutant. The Journal of heredity 95, 436–444. 15388771 10.1093/jhered/esh057

[CIT0007] Bielenberg DG, Wang Y, Li Z, Zhebentyayeva T, Fan S, Reighard GL, Scorza R, Abbott AG 2008 Sequencing and annotation of the evergrowing locus in peach [*Prunus persica* (L.) Batsch] reveals a cluster of six MADS-box transcription factors as candidate genes for regulation of terminal bud formation. Tree Genetics & Genomes 4, 495–507.

[CIT0008] Bordenave M, Goldberg R 1994 Immobilized and free apoplastic pectinmethylesterases in mung bean hypocotyl. Plant Physiology 106, 1151–1156. 12232398 10.1104/pp.106.3.1151 PMC159643

[CIT0009] Busov V, Carneros E, Yakovlev I 2016 EARLY BUD-BREAK1 (EBB1) defines a conserved mechanism for control of bud-break in woody perennials. Plant Signaling and Behavior 11, e1073873. 26317150 10.1080/15592324.2015.1073873 PMC4883858

[CIT0010] Cadman CS, Toorop PE, Hilhorst HW, Finch-Savage WE 2006 Gene expression profiles of Arabidopsis Cvi seeds during dormancy cycling indicate a common underlying dormancy control mechanism. The Plant Journal 46, 805–822. 16709196 10.1111/j.1365-313X.2006.02738.x

[CIT0011] Cai BD, Yin J, Hao YH, Li YN, Yuan BF, Feng YQ 2015 Profiling of phytohormones in rice under elevated cadmium concentration levels by magnetic solid-phase extraction coupled with liquid chromatography tandem mass spectrometry. Journal of Chromatography A 1406, 78–86. 26141271 10.1016/j.chroma.2015.06.046

[CIT0012] Castède S, Campoy JA, Le Dantec L, Quero-García J, Barreneche T, Wenden B, Dirlewanger E 2015 Mapping of candidate genes involved in bud dormancy and flowering time in sweet cherry (*Prunus avium*). PLoS ONE 10, e0143250. 26587668 10.1371/journal.pone.0143250 PMC4654497

[CIT0013] Chabikwa TG, Brewer PB, Beveridge CA 2019 Initial bud outgrowth occurs independent of auxin flow from out of buds. Plant Physiology 179, 55–65. 30404820 10.1104/pp.18.00519 PMC6324225

[CIT0014] Chen M, Tan Q, Sun M, Li D, Fu X, Chen X, Xiao W, Li L, Gao D 2016 Genome-wide identification of *WRKY* family genes in peach and analysis of *WRKY* expression during bud dormancy. Molecular Genetics and Genomics 291, 1319–1332. 26951048 10.1007/s00438-016-1171-6 PMC4875958

[CIT0015] Cole M, Chandler J, Weijers D, Jacobs B, Comelli P, Werr W 2009 *DORNRÖSCHEN* is a direct target of the auxin response factor MONOPTEROS in the *Arabidopsis* embryo. Development 136, 1643–1651. 19369397 10.1242/dev.032177

[CIT0016] Crawford S, Shinohara N, Sieberer T, Williamson L, George G, Hepworth J, Müller D, Domagalska MA, Leyser O 2010 Strigolactones enhance competition between shoot branches by dampening auxin transport. Development 137, 2905–2913. 20667910 10.1242/dev.051987

[CIT0017] De Lorenzo G, Ferrari S, Giovannoni M, Mattei B, Cervone F 2019 Cell wall traits that influence plant development, immunity, and bioconversion. The Plant Journal 97, 134–147. 30548980 10.1111/tpj.14196

[CIT0018] Dekkers BJ, Pearce S, van Bolderen-Veldkamp RP, et al 2013 Transcriptional dynamics of two seed compartments with opposing roles in Arabidopsis seed germination. Plant Physiology 163, 205–215. 23858430 10.1104/pp.113.223511 PMC3762641

[CIT0019] Devitt ML, Stafstrom JP 1995 Cell cycle regulation during growth-dormancy cycles in pea axillary buds. Plant Molecular Biology 29, 255–265. 7579177 10.1007/BF00043650

[CIT0020] Dewitte W, Scofield S, Alcasabas AA, et al 2007 Arabidopsis CYCD3 D-type cyclins link cell proliferation and endocycles and are rate-limiting for cytokinin responses. Proceedings of the National Academy of Sciences, USA 104, 14537–14542. 10.1073/pnas.0704166104 PMC1964848 17726100

[CIT0021] Dierck R, De Keyser E, De Riek J, Dhooghe E, Van Huylenbroeck J, Prinsen E, Van Der Straeten D 2016 Change in auxin and cytokinin levels coincides with altered expression of branching genes during axillary bud outgrowth in *Chrysanthemum.* PLoS ONE 11, e0161732. 27557329 10.1371/journal.pone.0161732 PMC4996534

[CIT0022] Domingo C, Roberts K, Stacey NJ, Connerton I, Ruíz-Teran F, McCann MC 1998 A pectate lyase from *Zinnia elegans* is auxin inducible. The Plant Journal 13, 17–28. 9680962 10.1046/j.1365-313x.1998.00002.x

[CIT0023] Endo A, Tatematsu K, Hanada K, et al 2012 Tissue-specific transcriptome analysis reveals cell wall metabolism, flavonol biosynthesis and defense responses are activated in the endosperm of germinating *Arabidopsis thaliana* seeds. Plant & Cell Physiology 53, 16–27. 22147073 10.1093/pcp/pcr171

[CIT0024] Ferguson BJ, Beveridge CA 2009 Roles for auxin, cytokinin, and strigolactone in regulating shoot branching. Plant Physiology 149, 1929–1944. 19218361 10.1104/pp.109.135475 PMC2663762

[CIT0025] Francis D, Sorrell DA 2001 The interface between the cell cycle and plant growth regulators: a mini review. Plant Growth Regulation 33, 1–12.

[CIT0026] Gillespie LM, Volaire FA 2017 Are winter and summer dormancy symmetrical seasonal adaptive strategies? The case of temperate herbaceous perennials. Annals of Botany 119, 311–323. 28087658 10.1093/aob/mcw264 PMC5314652

[CIT0027] Gordon SP, Heisler MG, Reddy GV, Ohno C, Das P, Meyerowitz EM 2007 Pattern formation during de novo assembly of the *Arabidopsis* shoot meristem. Development 134, 3539–3548. 17827180 10.1242/dev.010298

[CIT0028] Hawkesford MJ, De Kok LJ 2006 Managing sulphur metabolism in plants. Plant, Cell & Environment 29, 382–395. 10.1111/j.1365-3040.2005.01470.x 17080593

[CIT0029] Horvath DP, Anderson JV, Chao WS, Foley ME 2003 Knowing when to grow: signals regulating bud dormancy. Trends in Plant Science 8, 534–540. 14607098 10.1016/j.tplants.2003.09.013

[CIT0030] Horvath DP, Chao WS, Suttle JC, Thimmapuram J, Anderson JV 2008 Transcriptome analysis identifies novel responses and potential regulatory genes involved in seasonal dormancy transitions of leafy spurge (*Euphorbia esula* L.). BMC Genomics 9, 536. 19014493 10.1186/1471-2164-9-536 PMC2605480

[CIT0031] Lang GA 1987 Dormancy: a new universal terminology. Hortscience 22, 817–820.

[CIT0032] Lee Y, Karunakaran C, Lahlali R, Liu X, Tanino KK, Olsen JE 2017 Photoperiodic regulation of growth-dormancy cycling through induction of multiple bud-shoot barriers preventing water transport into the winter buds of Norway spruce. Frontiers in Plant Science 8, 2109. 29321789 10.3389/fpls.2017.02109 PMC5732187

[CIT0033] Maruyama-Nakashita A, Nakamura Y, Yamaya T, Takahashi H 2004 A novel regulatory pathway of sulfate uptake in *Arabidopsis* roots: implication of CRE1/WOL/AHK4-mediated cytokinin-dependent regulation. The Plant Journal 38, 779–789. 15144379 10.1111/j.1365-313X.2004.02079.x

[CIT0034] Maurya JP, Bhalerao RP 2017 Photoperiod- and temperature-mediated control of growth cessation and dormancy in trees: a molecular perspective. Annals of Botany 120, 351–360. 28605491 10.1093/aob/mcx061 PMC5591416

[CIT0035] Morris K, Linkies A, Müller K, Oracz K, Wang X, Lynn JR, Leubner-Metzger G, Finch-Savage WE 2011 Regulation of seed germination in the close Arabidopsis relative *Lepidium sativum*: a global tissue-specific transcript analysis. Plant Physiology 155, 1851–1870. 21321254 10.1104/pp.110.169706 PMC3091087

[CIT0036] Ni J, Zhao ML, Chen MS, Pan BZ, Tao YB, Xu ZF 2017 Comparative transcriptome analysis of axillary buds in response to the shoot branching regulators gibberellin A3 and 6-benzyladenine in *Jatropha curcas*. Scientific Reports 7, 11417. 28900192 10.1038/s41598-017-11588-0 PMC5595854

[CIT0037] Niu Q, Li J, Cai D, Qian M, Jia H, Bai S, Hussain S, Liu G, Teng Y, Zheng X 2016 Dormancy-associated MADS-box genes and microRNAs jointly control dormancy transition in pear (*Pyrus pyrifolia* white pear group) flower bud. Journal of Experimental Botany 67, 239–257. 26466664 10.1093/jxb/erv454 PMC4682432

[CIT0038] Pacifici E, Mambro RD, Ioio RD, Constantino P, Sabatini S 2018 Acidic cell elongation drives cell differentiation in the *Arabidopsis* root. The EMBO Journal 37, e99134. 30012836 10.15252/embj.201899134 PMC6092616

[CIT0039] Penfield S, Li Y, Gilday AD, Graham S, Graham IA 2006 *Arabidopsis* ABA INSENSITIVE4 regulates lipid mobilization in the embryo and reveals repression of seed germination by the endosperm. The Plant Cell 18, 1887–1899. 16844907 10.1105/tpc.106.041277 PMC1533976

[CIT0040] Rameau C, Bertheloot J, Leduc N, Andrieu B, Foucher F, Sakr S 2014 Multiple pathways regulate shoot branching. Frontiers in Plant Science 5, 741. 25628627 10.3389/fpls.2014.00741 PMC4292231

[CIT0041] Roman H, Girault T, Barbier F, et al 2016 Cytokinins are initial targets of light in the control of bud outgrowth. Plant Physiology 172, 489–509. 27462085 10.1104/pp.16.00530 PMC5074613

[CIT0042] Sakakibara H 2006 Cytokinins: activity, biosynthesis, and translocation. Annual Review of Plant Biology 57, 431–449. 10.1146/annurev.arplant.57.032905.105231 16669769

[CIT0043] Sarkar P, Bosneaga E, Auer M 2009 Plant cell walls throughout evolution: towards a molecular understanding of their design principles. Journal of Experimental Botany 60, 3615–3635. 19687127 10.1093/jxb/erp245

[CIT0044] Schaller GE, Street IH, Kieber JJ 2014 Cytokinin and the cell cycle. Current Opinion in Plant Biology 21, 7–15. 24994531 10.1016/j.pbi.2014.05.015

[CIT0045] Sechet J, Frey A, Effroy-Cuzzi D, et al 2016 Xyloglucan metabolism differentially impacts the cell wall characteristics of the endosperm and embryo during Arabidopsis seed germination. Plant Physiology 170, 1367–1380. 26826221 10.1104/pp.15.01312 PMC4775114

[CIT0046] Seeliger I, Frerichs A, Glowa D, Velo L, Comelli P, Chandler JW, Werr W 2016 The AP2-type transcription factors DORNRÖSCHEN and DORNRÖSCHEN‑LIKE promote G1/S transition. Molecular Genetics and Genomics 291, 1835–1849. 27277595 10.1007/s00438-016-1224-x

[CIT0047] Shen J, Zhang Y, Ge D, Wang Z, Song W, Gu R, Che G, Cheng Z, Liu R, Zhang X 2019 CsBRC1 inhibits axillary bud outgrowth by directly repressing the auxin efflux carrier *CsPIN3* in cucumber. Proceedings of the National Academy of Sciences, USA 116, 17105–17114. 10.1073/pnas.1907968116 PMC6708385 31391306

[CIT0048] Shim D, Ko JH, Kim WC, Wang Q, Keathley DE, Han KH 2014 A molecular framework for seasonal growth-dormancy regulation in perennial plants. Horticulture Research 1, 14059. 26504555 10.1038/hortres.2014.59 PMC4591672

[CIT0049] Shimizu-Sato S, Mori H 2001 Control of outgrowth and dormancy in axillary buds. Plant Physiology 127, 1405–1413. 11743082 PMC1540171

[CIT0050] Shinohara N, Taylor C, Leyser O 2013 Strigolactone can promote or inhibit shoot branching by triggering rapid depletion of the auxin efflux protein PIN1 from the plasma membrane. PLoS Biology 11, e1001474. 23382651 10.1371/journal.pbio.1001474 PMC3558495

[CIT0051] Skalák J, Vercruyssen L, Claeys H, et al 2019 Multifaceted activity of cytokinin in leaf development shapes its size and structure in Arabidopsis. The Plant Journal 97, 805–824. 30748050 10.1111/tpj.14285

[CIT0052] Sun MY, Fu XL, Tan QP, Liu L, Chen M, Zhu CY, Li L, Chen XD, Gao DS 2016 Analysis of basic leucine zipper genes and their expression during bud dormancy in peach (*Prunus persica*). Plant Physiology and Biochemistry 104, 54–70. 27107182 10.1016/j.plaphy.2016.03.004

[CIT0053] Takahashi H, Kopriva S, Giordano M, Saito K, Hell R 2011 Sulfur assimilation in photosynthetic organisms: molecular functions and regulations of transporters and assimilatory enzymes. Annual Review of Plant Biology 62, 157–184. 10.1146/annurev-arplant-042110-103921 21370978

[CIT0054] Tokunaga H, Kojima M, Kuroha T, Ishida T, Sugimoto K, Kiba T, Sakakibara H 2012 Arabidopsis lonely guy (LOG) multiple mutants reveal a central role of the LOG-dependent pathway in cytokinin activation. The Plant Journal 69, 355–365. 22059596 10.1111/j.1365-313X.2011.04795.x

[CIT0055] Trapnell C, Williams BA, Pertea G, Mortazavi A, Kwan G, van Baren MJ, Salzberg SL, Wold BJ, Pachter L 2010 Transcript assembly and quantification by RNA-Seq reveals unannotated transcripts and isoform switching during cell differentiation. Nature Biotechnology 28, 511–515. 10.1038/nbt.1621 PMC3146043 20436464

[CIT0056] Velappan Y, Signorelli S, Considine MJ 2017 Cell cycle arrest in plants: what distinguishes quiescence, dormancy and differentiated G1? Annals of Botany 120, 495–509. 28981580 10.1093/aob/mcx082 PMC5737280

[CIT0057] Vergara R, Noriega X, Aravena K, Prieto H, Pérez FJ 2017 ABA represses the expression of cell cycle genes and may modulate the development of endodormancy in grapevine buds. Frontiers in Plant Science 8, 812. 28579998 10.3389/fpls.2017.00812 PMC5437152

[CIT0058] Waldie T, Leyser O 2018 Cytokinin targets auxin transport to promote shoot branching. Plant Physiology 177, 803–818. 29717021 10.1104/pp.17.01691 PMC6001322

[CIT0059] Wisniewski M, Norelli J, Artlip T 2015 Overexpression of a peach CBF gene in apple: a model for understanding the integration of growth, dormancy, and cold hardiness in woody plants. Frontiers in Plant Science 6, 85. 25774159 10.3389/fpls.2015.00085 PMC4343015

[CIT0060] Wybouw B, De Rybel B 2019 Cytokinin – a developing story. Trends in Plant Science 24, 177–185. 30446307 10.1016/j.tplants.2018.10.012

[CIT0061] Yamane H, Ooka T, Jotatsu H, Hosaka Y, Sasaki R, Tao R 2011 Expressional regulation of *PpDAM5* and *PpDAM6*, peach (*Prunus persica*) dormancy-associated MADS-box genes, by low temperature and dormancy-breaking reagent treatment. Journal of Experimental Botany 62, 3481–3488. 21378115 10.1093/jxb/err028 PMC3130173

[CIT0062] Yordanov YS, Ma C, Strauss SH, Busov VB 2014 EARLY BUD-BREAK 1 (*EBB1*) is a regulator of release from seasonal dormancy in poplar trees. Proceedings of the National Academy of Sciences, USA 111, 10001–10006. 10.1073/pnas.1405621111 PMC4103365 24951507

[CIT0063] Zhang K, Diederich L, John PC 2005 The cytokinin requirement for cell division in cultured *Nicotiana plumbaginifolia* cells can be satisfied by yeast Cdc25 protein tyrosine phosphatase: implications for mechanisms of cytokinin response and plant development. Plant Physiology 137, 308–316. 15618425 10.1104/pp.104.051938 PMC548861

[CIT0064] Zhao K, Zhou Y, Ahmad S, Xu Z, Li Y, Yang W, Cheng T, Wang J, Zhang Q 2018 *a* Comprehensive cloning of Prunus mume dormancy associated MADS-box genes and their response in flower bud development and dormancy. Frontiers in Plant Science 9, 17. 29449849 10.3389/fpls.2018.00017 PMC5800298

[CIT0065] Zhao X, Wang Q, Li C, Chen X, Xiao W, Gao D, Fu X 2018 *b* Genome-wide identification of ethylene responsive factor (ERF) family genes in peach and screening of genes related to germination. Chinese Bulletin of Botany 53, 612–624.

