# Supplementary material for: EARLY BUD BREAK 1 triggers bud break in peach trees by regulating hormone metabolism, the cell cycle, and cell wall modifications
Source: J Exp Bot. 2020 Mar 9;71(12):3512–23. doi: 10.1093/jxb/eraa119 (PMC7475240; doi:10.1093/jxb/eraa119)
Supplement: eraa119_suppl_Supplementary_Material [file eraa119_suppl_supplementary_material.pdf]

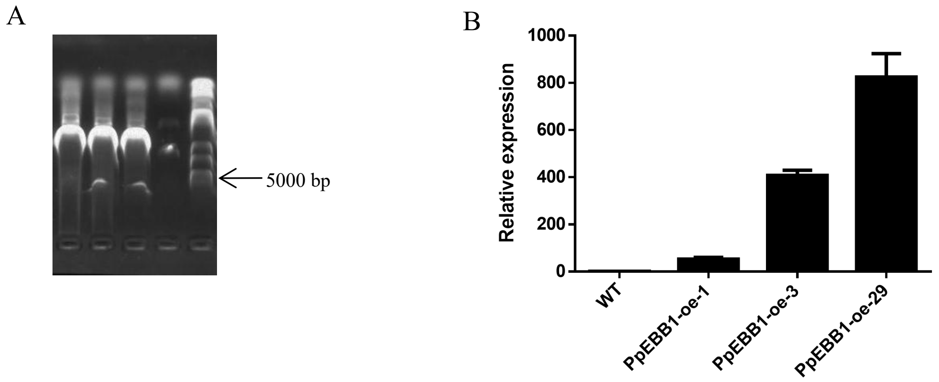

Fig.S1 Result of PCR indicated *PpEBB1* was transferred into poplar successfully, strips from left to right were *PpEBB1-oe-1*, *PpEBB1-oe-3*, *PpEBB1-oe-29*, WT, and marker respectively (A). The expression level of *PpEBB1* in WT, *PpEBB1-oe-1*, *PpEBB1-oe-3*, *PpEBB1-oe-29* lines (B).

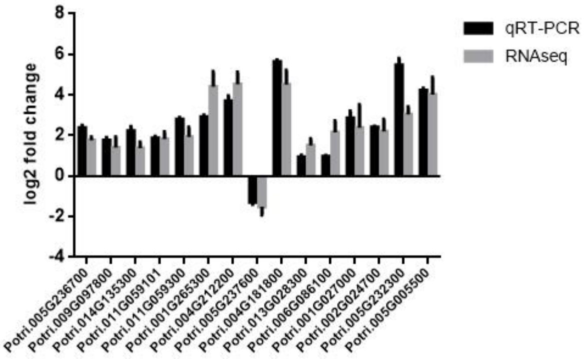

Fig. S2 Verification of RNA-seq results used qRT-PCR. The expression and tendency of different genes were similar with the result of RNA-seq, confirming that RNA-seq result was credible.

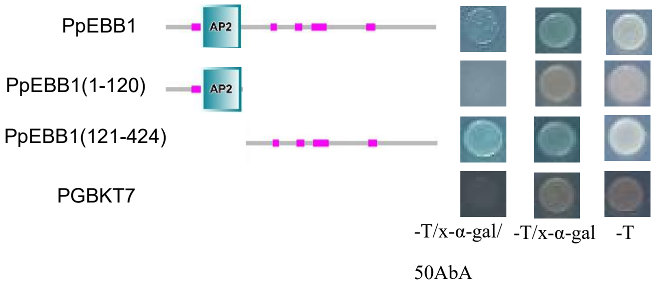

Fig.S3 Auto-activation verification of full length of PpEBB1, PpEBB1 1-120aa, and PpEBB1 120-424aa used Y2H assay.

Table S1. Primers used for qRT-PCR in this study.

Abbreviated name	Forward primer	Reverse primer

LOG1	TTATTGCCTTGCCTGGTG	CCTTCCTCTACTGCTTTGTCAA
LOG2	GCACCAAAGAAAGGCAGA	CGAACAAGGCAAGCAAAC
AHP6	CGTATCTGAAGTTGTCACCATC	CCCAGCACGGTTGTTT
CKX5	GATGAATCCACTGGCGAT	ACAAACCCTTGGCTCTCA
AHP2.1	TTCAAGATGAGAGCAACCC	GCTACCCTTCAACTGGTGA
LOG6	GCTGAAATGGCTCGTCAA	GCTGGTGTGATGAATCCTTC
ARR8	CTTATCAAGTCACGGCAGTG	CATAGCCTGTCATTCCTGG
ARF5	ACCTGGACATTTCGCCAT	GTTGTTTGTTGACGGTTTGC
ARF6	GAAGGCAATCAACTCGGA	GGCACAATAACTGAGAAGGC
ARF3	TCCTGGTTCCCACAGTTCT	CACGAGCCTCATTAGCATTT
ARF3	GCAAGTGATGAGGTGTATGC	TGTCAGAAGCAGTTAGGGTCT
ARF2	GATTCTCTGTTCCAAAGCGA	TGTGCTCCAACCACTCGTA
CYCD3.3	CCTCTTTGTTTGCCAAGG	TTTCTGAAGGTGGACACTGA
LOG7	GCTGAAATGGCTCGTCAA	ACAAACCCTTGGCTCTCA
CYCD3.2	CCATTGACTACCAGACCCA	AGTGACGAAGAGACCGATG
EXPA3	GGAGTGAACACAGCAGCAT	CAAAGTTAGGAGGGCAGAAA
LOG5	CACAGGAGAAACAGTTGGAGA	TTGAGCAAACCCACAGGT
EXPA8	CCCTCCAGCACTTTGACAT	CACTGAATGAACATCTCCTGC
CKX4	TGTCACAGACCAAGAGCGT	CATCCGATTGAAACTGGC
AHP5	AGAGCCCTCAGTTTGTGATT	CCTGTCCTTGTCCTTGTCTT
AHP2	GCAACCCAGATTTCGTAGTT	TATTTCGCTCCTCGCAGT
YUC6	GGTCCAATCCAACGAGTCT	TCCACAGCCTACAACCAAA
PIN1	CCAAGGTTCCATTACCACG	AGGTCCCTACCATCTTCAGC
PIN10	TTATGGCAATGACCTCGG	CAGCACTCTTTGGGTGTAGC
PILS5	GATACAGAAAGCCGTGCTATTG	TGCTCCAAAGATGAATCCC
PILS3	AAGCAGCACACCATTTCG	AAGGTTGACCAAAGGGTAAGAG
PILS1	CATCTTCATTTCCGAGCG	CGAACCACCATCTCATCAG
AUX4	GGGTCTGTCTATGATGCCTG	CCTTTCTGCTTCGGTATTCA
AUX3	CAGGTTGCTCAGGTTCTGTT	CCTTTCTGCTTCGGTATTCA
AUX1	CACATTTGGGTTTGCGTG	ACGGCTGAGTTGATAGGTCC
SAUR69	ATGCGACGAGGCTGTCTTT	GCAACGGTCTTGATTCACC
SAUR61	ATGGCAAGGAAGTGGCA	AAGACTGCTGGCGAGTTTG
SAUR6	TGATGGCTATTCGCTTGC	GTAGGTCCTGAAATGAAGGTTG
SAUR55	GGAGGTGGTATGTCTGGTTG	GTCTTGATGTTCTCTTGCTTCC
SAUR38	ATGTCAGCCAATCGCATAC	AAAGCCAAACTCTTCCTCG
SAUR34	AACGCAGAAGAGAACCAGC	CACAGCCAAGTGTCCTTTG
SAUR22	TAACTGGAAGCCTTGCCA	GTCAAACCACCCATTGGA
SAUR23	GTGAGGAGCAACAAAGGTTT	GTGGTGGTGATACTGGGAA
SAUR14	TGATGGCTATTCGCTTGC	CTTGAAATGAAGGCTGGTTC
GH3.6.1	CCTGTTGATGCCTGTTATGAGT	TGTCCTTGAAATGGGTGCT
GH3.9	CGGAAATGTCTACTCCCTGTG	GCCTTAGGACTTGATGACGG
GH3.17	GCCTGTTGATGCCTGTTATG	TGTCCTTGAAATGGGTGCT
GH3.6	TGTGTTGAGAGTGGTTGGG	GGAATGGTAGTGGTGTCTGC
IAA33	CGAAGGCTCTAAGGCAGAT	AAGGTCACCAGCAAGAAGG

IAA16	TTCCATCTTCCGCTGAGA	GCAACTCTACCACCGCTTT
IAA19	GCAAACAGCACAACCGAT	GCTCCGTCCATACTAACTTTCA
IAA18	TGTCCCATTTCCTGCTACC	GGAGGTTTATTTCGTGACTCAG
IAA12	TTTCCTCTGCTTGCGTTG	CAGCCTTTGCTTGGTTCA
IAA4	TGATGAGCCAGAGAAACCA	TCCCACTACTTGTGCCTTG
IAA6	TGGCACAACCTCTTGGACT	CGATACGAACAAACTGGTGG
CDKB2.1	CAACCACCACTCTTCGTGA	GGAATGTTCTCTCCAGTTTGG
CYCA1.2	ACAGAGAATGTAGGCAAGGC	CTATTAGAAGCAGGAGGTCCC
CYCB1.1	GTGGATTGGTTGGTTGATGT	CATTTACCTCAGGAGCCCA
CYCB1.3	GCAATCCCAACCAGACATC	GAAGTTCCCTCCTTGTTACCAT
CYCD1.1	GCTCATCAAGACACGCTGA	GCTACAGATAGGCAGGCTACTG
CYCD2.1	CACCATCACCATCCATCAT	ACTCGTAGTGGGCATACACC
CYCD6.1	GATGAAGAACGCCCACTT	GAGGTTGTGAGGATGATGG
CYCP2.1	GGTTGGAGGAGTGAGCAAT	TCTCTGTGACGCACCATTTA
EXPB3	TAGTGGATGTGAAGCCATTG	ACTGAGGTCAAAGTGGGTGT
GH17.3	TCATCCTCATCACTGTCGG	CAGAAGAAGGTGGCTCAGAC
GH17.4	CACACTCATTGACAAGGTGAAG	GGCAGGAATAAAGGGAACC
GH17.5	TCATCCTCATCACTGTCGG	CAGAAGAAGGTGGCTCAGAC
GH17.6	AGGCTCTTACCAGCAATGC	GCACGACTAAACTCCAACAAG
GH9B18	TCTTCTCGCCACACTACTCA	CCATCCATCACTTAGCCCT
XTH28	AGTGCTTCTATCAAGTTGCCAG	AGTGCTGCCATTGCCATA
XTH31	ACTATCCTGGCTCCCGAAT	TGATGGCAGCACCAAAGTA
XTH9	CAGATGATTGGGCTACACAA	CACCACTGCTGCTACATTTC
CSLD5	TGTGGTGACTGGTTATCGTATG	GGCGAGTAGCAAATAGAGCAT
CSLE1.1	GATGATGGAGTTTGGAGCC	GATACAGAGGCAAGTTGACGA
CSLE1.2	CCAGATTGAAGCAACCACA	TTGTCCATAGCCTGAGGGT
CSLE1.3	TGGGTTGGTTGAAGTTGG	CAGACAAAGTTGAGGGATGG
CEL3	ATGAAAGCAACCAGGCAC	TGATGGCAAAGAAGAACCTC
CEL5	GGTGGCTGTCATAGACTTCCT	AGAGTGCTTCCTTACGGCA
CALS1	TGGAAATGGAGAGCAGACTT	TGAGAACAAGATAGAGGCGG
PLL1	TTTCTGATGACACCCAGGC	GGTCCCAGTTCTTAGGCTCA
PLL2	TTGCCTCCTCACCTTTAGC	GACCCTGAAACCTCACTTGA
PLL3	GGCTACGGGCATAACTTGT	TTACATTCCCAGACCCTCC
PLL5	ATGGCACAACACTTCCTGG	GCTGGTGATTTGATGCTAAGC
PLL6	TCTCATTTCACTACCACCTGC	TCTGTGTCACCATCACCAAC
PME3	GGATGGTTTCTCTCACGATG	GCCACACTCTTTCATTACCTTC
PME1	GGAGGAGGCACTACAACTTTC	TGAGAATGGACATAGAGGGTG
PME32	TCCTTGACATCGTTTCCG	TCCATCCTTAGCCACAACA
PME33	CCGAGTCAATGAGTGATGTG	TCTTCTCCAACGAATGTGTG
PAE12	TGCTGCTGCTGTTCTTTG	GCTGATTTGATGAGAGTGAGG
PAE11	TTGCTCAAGGCAGAAGGAG	CACAGGACTCAACACTCTCACA
PtActin	GATTTGTCCCTCGCGCTGT	TCGGTATAATGACCCTTGGCC
PpActin	GTTATTCTTCATCGGCGTCTTCG	CTTCACCATTCCAGTTCCATTGTC
PpEBB1	CCAAGAAGTCAATGGACCCT	TGTGGTTGTGGCAGCAAT

Table S2. Genes may interacted with PpEBB1 screened by Y2H.

Gene name	Gene description in JGI
Prupe.8G258100	Prunus persica heat shock factor-binding protein 1
Prupe.8G098000	glucan endo-1,3-beta-glucosidase 7
Prupe.2G296500	cytochrome P450 89A2
Prupe.4G029700	Prunus persica protein heat-stress-associated 32
Prupe.6G063300	proliferating cell nuclear antigen
Prupe.6G231300	SUMO-conjugating enzyme SCE1
Prupe.7G034500	dual specificity protein phosphatase 12
Prupe.5G057900	Prunus persica expansin-like B1
Prupe.2G183100	ubiquitin-conjugating enzyme E2-17 kDa
Prupe.4G056500	E3 ubiquitin-protein ligase
